# Can River Chief System Policy Improve Enterprises’ Energy Efficiency? Evidence from China

**DOI:** 10.3390/ijerph20042882

**Published:** 2023-02-07

**Authors:** Da Gao, Chang Liu, Xinyan Wei, Yang Liu

**Affiliations:** 1 School of Law and Business, Wuhan Institute of Technology, Wuhan 430200, China; 2 School of Economics, Huazhong University of Science and Technology, Wuhan 430070, China; 3 School of Economics and Management, Huazhong Agricultural University, Wuhan 430070, China

**Keywords:** environmental regulation, river chief system, green total factor energy efficiency, difference-in-differences, mechanism tests

## Abstract

The river chief system (RCS) is an autonomous environmental policy implemented by local governments in China that incorporates environmental responsibilities into the performance evaluation. Although existing literature suggests that RCS can reduce water pollution, the impact of RCS on energy efficiency has not been assessed. Therefore, this paper compiles data on industrial enterprises and industrial pollution in China from 2003 to 2013 and empirically examines the impact of RCS on green total factor energy efficiency (GTFEE) by using a multiple difference-in-difference approach. The results show that RCS significantly enhances firms’ GTFEE, and a series of tests confirm the robustness of the findings. Second, we further explore how RCS affects GTFEE, the mechanism tests conclude that the RCS improves GTFEE mainly through optimizing energy structure and promoting technological innovation. Third, compared with small firms, exporters, and firms in non-heavy polluting industries, the RCS has a greater effect on improving the GTFEE of large firms, non-exporters, and firms in heavily polluting industries. This study provides new and novel ideas for emerging countries to improve environmental policies and achieve sustainable development.

## 1. Introduction

As an important factor driving economic development, energy is becoming increasingly important in the industrialization of countries [1]. However, carbon dioxide emission caused by massive energy consumption has become the primary factor of global warming [2]. As the world’s largest developing country, China’s energy consumption in 2021 accounted for 26.11% of the world’s total energy consumption, and its total carbon emissions accounted for 30.69% of the world’s total carbon emissions [3]. Therefore, the twin carbon plans of peak carbon by 2030 and carbon neutrality by 2060 pledged by Chinese leader Xi Jinping at the “Waiting Ambition Summit” have attracted much global attention. Energy efficiency refers to the economic and environmental benefits obtained from a unit of energy consumption [4]. Improving energy efficiency is important to promote economic growth and reduce greenhouse gas emissions [5]. Therefore, it is of great theoretical value and policy guidance to seek ways to improve energy efficiency.

Due to the externalities of improving energy efficiency and solving environmental pollution, environmental regulation has become the first choice for the government to improve energy efficiency [6]. Although the central government of China has introduced a series of emission reduction policies and strengthened investment in environmental protection, the improvement of energy efficiency in China is lagging in the world [7]. Most scholars agree that the failure of local governments to implement environmental policies formulated by the central government effectively is the cause of ineffective environmental governance in the Chinese government [8]. Specifically, environmental policies in China are generally “top-down,” with the central government formulating environmental policies and local governments primarily implementing them, but the responsibility for environmental governance is not included in the promotion and assessment of local officials. As a result, local governments often do not pay as much attention to environmental protection as they do to economic development, which ultimately leads to poor implementation of central environmental policies [9,10].

Therefore, the river chief system (RCS), as a powerful means of local environmental regulation, came into being. The RCS is a major innovative environmental protection policy independently implemented by local governments in China to promote water ecological and environmental governance. In 2007, the Wuxi city government took the lead in implementing the river chief system in response to the cyanobacteria crisis in Taihu Lake. Several local governments have since copied the policy. By 2018, the RCS had been fully implemented nationwide in China [11]. Unlike other environmental protection policies in China, the RCS is independently proposed by local governments, which for the first time incorporates environmental protection responsibility into the assessment and promotion of local officials [11]. In this case, local officials cannot ignore environmental pollution problems based on the pressure of assessment and promotion, so they will naturally intensify environmental protection efforts and increase environmental governance expenditure [8]. In view of this, this paper attempts to explore the relationship between RCS, a bottom-up environmental policy developed by local governments, and energy efficiency.

However, there is little literature examining the impact of the RCS on energy efficiency. To fill this research gap, we empirically examine the impact of the RCS on energy efficiency using a multiple difference-in-differences (DID) methodology. First, we measured firms’ green total factor energy efficiency (GTFEE) based on relevant data from Chinese industrial firms. Second, we take the establishment of the river chief system (RCS) by local governments as a quasi-natural experiment. We find that the river chief system (RCS) significantly improved the GTFEE of firms. At the same time, this paper provides two ways to explore the RCS improves firms’ GTFEE mainly through two aspects: improving energy structure and enhancing technological innovation. In addition, there is heterogeneity in the policy effects of the RCS. Specifically, RCS has a greater effect on improving the GTFEE of large firms, non-exporting firms, and firms in heavily polluting industries than small firms, exporting firms, and non-heavy polluting industries.

The main contributions of this paper are as follows. First, to the best of our knowledge, this paper is the first to measure GTFEE at the firm level. For energy efficiency at the firm level, the existing literature mainly calculates the single factor energy efficiency (SFEE) at the firm level [12,13], but the GTFEE at the firm level was not measured. Compared with SFEE at the enterprise level, GTFEE at the enterprise level takes into account the mutual substitution of different production factors and includes undesired output, which can reflect the energy economic system efficiency of enterprises more comprehensively and effectively [14,15]. Second, this paper extends the research on environmental regulation and energy efficiency. The existing literature mainly examines the impact of environmental regulations on energy efficiency from two perspectives: market-oriented regulations [16,17] and “top-down” command-and-control regulations [18,19] to examine the impact of environmental regulation on energy efficiency. This paper is the first to examine the impact of environmental regulations on energy efficiency from the perspective of “bottom-up” command-and-control regulations using the river chief system as a policy, filling a gap in the existing literature. Third, we further explore the potential impact mechanism of RCS on enterprise GTFEE and analyze and confirm that RCS can improve GTFEE through two important channels: improving energy mix and enhancing technological innovation, the latter effectively verifying the existence of the “Porter effect”.

The rest of the paper is structured as follows. Section 2 reviews the relevant literature. Section 3 summarizes the background of the river chief system and presents the research hypothesis. Section 4 provides an introduction to the methodology, variables, and data. Section 5 presents the empirical results of the paper. Section 6 presents the further analysis. Section 7 presents conclusions and implications.

## 2. Literature Review

There are three branches of literature relevant to our research. The first branch related to this paper is the measurement of energy efficiency. The second branch is the impact of environmental regulation on energy efficiency. The third branch is mainly concerned with evaluating the effects of the RCS.

### 2.1. The Measurement of Energy Efficiency

The measurement of energy efficiency can be divided into two types: single factor energy efficiency (SFEE) and total factor energy efficiency (TFEE). SFEE is usually defined as the ratio of desired output to energy inputs, such as energy consumption per unit of GDP [20]. Although SFEE is simple to calculate and easy to understand, it only considers energy input and ignores the mutual substitution between different production factors [21]. Hu and Wang [22] proposed total factor energy efficiency (TFEE) for the first time. TFEE takes energy, capital, labor, and other production factors as input factors, effectively overcoming the deficiency of SFEE. However, the calculation of TFEE does not include undesired output, so it also has deficiencies [23]. Some scholars began to incorporate pollutant emissions into the calculation of total factor energy efficiency and defined it as green total factor energy efficiency (GTFEE) [24]. Compared with SFEE and TFEE, GTFEE can more comprehensively and effectively reflect the efficiency of the economic energy system [25,26].

Existing studies on energy efficiency measurement mainly focus on the macro-regional level, and the measurement of energy efficiency at the enterprise level is less. For example, Zhou et al. [27] used the DEA method to assess the TFEE and its influencing factors in different member countries of the Regional Comprehensive Economic Partnership (RCEP). Cheng et al. [20] calculated the TFEE of 30 provinces in China from 1997 to 2016 and found that the TFEE in eastern China was the largest, followed by the central and western regions. Wu et al. [14] used Chinese provincial panel data from 2006 to 2017 and empirically found that Internet development can improve local GTFEE. Using city-level data, Liu et al. [28] empirically found that digital finance has a significant positive impact on urban GTFEE. Based on the panel data of Chinese cities from 2003 to 2016, Hong et al. [17] empirically found that the carbon emission trading system could improve the SFEE and TFEE of cities through green innovation and resource allocation channels. Feng et al. [29] found that urbanization has a significant inhibitory effect on urban TFEE. For the study of enterprise energy efficiency, Huang et al. [12] empirically found that using robots in production could significantly improve the SFEE of enterprises based on the data of Chinese enterprises from 2001 to 2012. Bu et al. [13] measured the SFEE of Chinese industrial enterprises and empirically found that Environmental Information Disclosure (EID) significantly improved the SFEE of enterprises. It can be seen that there is a lack of indicators to measure the energy efficiency of enterprises in existing studies, and only a few research have calculated SFEE in firms, and there is no measurement of GTFEE at the enterprise level.

### 2.2. The Impact of Environmental Regulation on Energy Efficiency

There are mainly two types of environmental regulations: market-oriented and command-and-control [17] Market-oriented regulations mainly include pollution emission trading systems and environmental tax, which is an environmental management method to internalize environmental costs into the production function of enterprises. Command-and-control regulations mainly refer to an environmental management method in which the government sets specific environmental emission standards to achieve pollution control targets [30]. Meanwhile, command-and-control regulations can be divided into two types: “top-down” and “bottom-up” [31]. “Top-down” environmental policy means that the central government promulgates policies and regulations, and ministries and local governments are responsible for implementing the initiatives that advance them. “Bottom-up” environmental policies are implemented spontaneously by local governments.

For the relationship between market-oriented regulations and energy efficiency, most studies have shown that market-oriented regulations can significantly improve TFEE [16,17]. Regarding the relationship between command-and-control regulations and energy efficiency, some studies concluded that command-and-control regulations are very helpful in improving the energy efficiency of cities [15,19]. Some scholars also argue that command-and-control regulations are ineffective in improving energy efficiency [18]. However, the above command-and-control regulations are “top-down” types, and few scholars have studied the impact of “bottom-up” types of command-and-control regulations on energy efficiency.

### 2.3. The Effects of the RCS

Since the implementation of the RCS, many scholars have conducted theoretical and empirical analyses of its effects on society’s economy. 

Most of the existing studies focus on the effect of RCS on wastewater treatment, and it is concluded that it has a significant effect on wastewater treatment. From the perspective of theoretical research, Zhang et al. [32] proposed that the RCS is integrated into the traditional Chinese environmental governance hierarchy through institutional embeddedness. Li et al. [11] found that RCS has a different impact on different water pollutants and is not as effective as the government claims. From the perspective of empirical research, Xu et al. [33] developed a differential game model under stochastic disturbance factors and found that the average effect of water pollution control was greater under RCS than under non-RCS. Wang et al. [34] also used a game model to conclude that RCS implemented by local governments would induce companies to treat wastewater and accelerate the level of environmental management. Li et al. [30] empirically investigated the effect of the “river chief system” on water pollution control using a regression discontinuity method. The results showed that the “river chief system” policy had a positive effect on river pollution control. Ouyang et al. [35] used a DID approach and found that RCS significantly reduces the amount of wastewater discharged per unit of GDP. The long-term effectiveness of this effect is based on the fact that RCS can promote the upgrading of local industrial structures.

In addition to water pollution, a few scholars have also studied the effects of RCS on other aspects. For example, Wang et al. [36], based on city-level data from the Yangtze River Economic Zone in China, found empirically that RCS increased overall SDG indicators, particularly innovation, education, and consumption levels. A study by Xu et al. [37] found that RCS significantly increased the profits of polluting firms by 3.1%. The increase in earnings in the heavily polluting industries is mainly due to the significant increase in market concentration and the possible transfer of adverse RCS shocks along the production line. However, no literature has examined the impact of the RCS on energy efficiency.

## 3. Background and Research Hypothesis

### 3.1. The Background

China’s rapid economic growth has been accompanied by severe environmental damage [38]. Recognizing the serious environmental problems in China, the Chinese government has made environmental protection a basic state policy in China since the early 1980s. It has enacted a series of laws and regulations to combat environmental pollution. In 2007, a large-scale cyanobacteria crisis broke out in the Taihu Lake area of Wuxi, Jiangsu Province, causing water supply problems and severe social impacts on the city. In response to the cyanobacteria crisis in Taihu Lake, Wuxi was the first city to introduce and implement the river chief system (RCS) in 2007. The first component of the RCS is the appointment of responsible persons (usually the main party and government officials at all levels) for the pollution control of each river and linking the pollution control to the political performance of officials. In China, rapid economic growth favors the promotion of key local leaders. Similarly, after the implementation of RCS, if the river chief can effectively control water pollution, then the river chief can also be promoted faster [11]. This environmental policy relating pollution management to officials’ performance appraisal provides a strong incentive for local officials to control pollution [39]. The second component of the RCS is the establishment of a regular joint meeting where the river chief coordinates conflicts of interest between different sectors such as the agricultural sector, the environmental sector, and the water sector. The RCS has achieved significant results in preventing water pollution in a short period, and the policy innovation of the RCS quickly spread from Wuxi to other provinces and cities in China. The implementation schedule of the river chief system across the Yangtze River Delta is detailed in Table 1.

### 3.2. Research Hypothesis

The failure of local governments to effectively implement the environmental policies formulated by the central government is the reason for the inefficient environmental governance of the Chinese government [8]. Specifically, China’s environmental policies are generally “top-down”, with the central government as the maker of environmental policies and the local governments as the main implementers of environmental policies. Local governments have room for discretion in implementing environmental policies, while economic growth is still the main indicator of performance evaluation for local officials. Therefore, local governments prioritize local economic development and ignore environmental pollution [9,10]. Ultimately, such top-down environmental policies from the central government tend not to work well.

The RCS is an environmental policy to manage water pollution but also impacts economic and other environmental pollution [36]. First, the RCS clarifies the responsibility of specific managers for water environmental protection and establishes a unified environmental protection agency to coordinate possible conflicts of interest in environmental protection between regions or sectors [35,40]. This process is conducive to improving the working mechanism of local government environmental law enforcement and strengthening the assessment and accountability of other environmental pollution-related indicators [8]. Strict environmental regulation by local governments is bound to increase production costs and force companies to improve energy efficiency. Secondly, the RCS encourages the public to become supervisors of environmental water management and actively participate in environmental governance [35,40]. Public supervision can force enterprises to invest more in the environment, improving their energy efficiency of enterprises [13]. Based on the above analysis, we propose the following hypotheses:

**Hypothesis** **1.** *The RCS can significantly improve enterprises’ green total factor energy efficiency.*

The RCS strengthens the assessment accountability of environmental pollution and increases the environmental regulation of enterprises by citizens [36]. Firms trade-off the consumption of low-carbon clean energy and high-polluting energy in the production process, and the increase in emission costs motivates firms to choose to use low-polluting and low-energy clean energy instead of high-polluting and high-energy energy, and eventually firms’ energy mix is optimized. Most empirical studies also support that environmental regulation can optimize the energy mix. For example, with the data on China’s primary energy consumption varieties, Shi et al. [41] found that the level of environmental regulation was significantly and negatively related to the scale of coal consumption. Bu et al. [13], using firm-level data in China, found that environmental information disclosure (EID) can increase the use of fuel oil and clean gas while reducing coal consumption by firms. At the same time, it has become a consensus in academia that improvements in the energy mix can increase energy efficiency. For example, using Chinese data, Han et al. [42] found that a shift in China’s energy mix from coal to oil, hydropower, and nuclear power can effectively improve total energy efficiency. Further, Bilgen [43] stated tha the improvement of energy efficiency by energy mix is mainly through substituting energy-intensive energy sources. Based on the above analysis, we propose H2 as follows.

**Hypothesis** **2.** *The RCS improves firms’ GTFEE by optimizing energy structure.*

According to Porter’s hypothesis, environmental regulation can produce “innovation compensation effects”. Specifically, appropriate environmental regulation can force firms to engage in green innovation activities to reduce production costs and improve firm competitiveness through advanced green technologies to compensate for the additional costs of environmental regulation [44]. First, RCS strengthens environmental regulation by the government and imposes additional environmental compliance costs on firms. Firms increase their R&D on green technologies to reduce environmental compliance costs [15]. Second, RCS links pollution control to officials’ performance appraisals. As a result, local governments will strengthen their support for enterprises to conduct green innovation activities such as energy conservation and emission reduction, including various tax and talent incentives [8].

On the other hand, technological innovation is an important way for environmental regulation to improve GTFEE [15]. Many studies point out that for a given output, green technology innovations reduce energy factor inputs and replace them with other factors of production, thereby increasing GTFEE [45,46]. Specifically, based on data from Italian foundries, Cagno et al. [45] found that the more innovative a company is, the more willing it is to adopt energy-efficient technologies and the greater the improvement in energy efficiency. Research by Aldieri et al. [46] shows that innovations in clean technology can improve energy efficiency. Based on the above analysis, we propose H3 as follows.

**Hypothesis** **3.** *The RCS improves firms’ GTFEE by improving technological innovation.*

## 4. Methodology and Data

### 4.1. Methodology

Referring to the research of Ouyang et al. [35] and Li et al. [11], we regard the implementation of the river chief system (RCS) in cities as a quasi-natural experiment and use a staggered difference-in-differences (DID) methodology to test the impact of RCS on firms’ GTFEE. The DID methodology is an econometric method for estimating causal effects. Its basic idea is to regard public policy as a natural experiment to evaluate the net impact of a policy. Specifically, all samples are first divided into two groups, one group is affected by the policy, that is, the experimental group; the other group is not affected by the policy, that is, the control group. Then, I net impact of the policy is then obtained based on the difference in the change between the experimental and control groups before and after the policy is implemented. The specific model is as follows:

(1) $$GTFEE_{ijkt}=\alpha +\beta RCS_{it}+\gamma X_{ijkt}+\delta_{i}+\delta_{j}+\delta_{k}+\delta_{t}+\varepsilon_{ijkt}$$

In Equation (1), $GTFEE_{ijkt}$ represents *GTFEE* of firm *k* in industry *j* in ciIy *i* in year *t*. $RCS_{it}$ is the dummy variable. We set RCS to 1 if the cIty *i* has implemented RCS in year *t* and 0 otherwise. The coefficient β suggests the impact of RCS on the GTFEE of firms. If β is positive and significant, it suggests that RCS can improve the GTFEE of firms. $X_{ijkt}$ is a series of firm-level control variables. $\delta_{i}$, $\delta_{j}$, $\delta_{k}$, and $\delta_{t}$ represent city fixed effect, industry fixed effect, firm fixed effect, and year fixed effect, respectively. Among them, city fixed effects $\delta_{i}$ are measures of unique characteristics of each city that do not vary over time. Industry fixed effects $\delta_{j}$ are measures of unique characteristics of each industry that do not vary over time. Firm fixed effects $\delta_{k}$ are measures of unique characteristics of each firm that do not vary over time, and year fixed effects $\delta_{t}$ are measures of unique characteristics of each year that do not vary with other factors, such as macroeconomics. $\varepsilon_{ijkt}$ is an error term. All standard errors are clustered at the firm level.

An important premise of difference-in-difference estimation is that the samples of the experimental and control groups share a common trend of change before the implementation of the policy event. Therefore, the observed differences between the two sample groups are fragmented due to policy treatment effects. To ensure the validity of the DID model, referring to Beck et al. [47] we next construct the following model to verify whether the samples satisfy the parallel trend assumption.

(2) $$GTFEE_{ijkt}=\alpha +\sum_{m=-4}^{m=3}\beta_{m}RCS_{i,t+m}+\gamma X_{ijkt}+\delta_{i}+\delta_{j}+\delta_{k}+\delta_{t}+\varepsilon_{ijkt}$$

In Equation (2), $RCS_{i,t+m}$ is a series of dummy variables that equals 1 when there are m years away from the implementation of RCS in city *i*. For example, when *m* = 2, the dummy variable $RCS_{i,t+2}$ indicates that city *i* implemented RCS in year *t* + 2, which estimates the effect in the second year after the implementation of RCS. Therefore, $RCS_{i,t+2}$ = 1 in the second year after RCS implementation, and $RCS_{i,t+2}$ = 0 in other years. Similarly, when *m* = −1, the dummy variable $RCS_{i,t-1}$ indicates that city *i* implemented RCS in year *t* − 1, which estimates the effect in the first year before RCS implementation. Therefore, $RCS_{i,t-1}$ = 1 in the first year before RCS implementation, and $RCS_{i,t-1}$ = 0 in other years. We set the previous year of RCS implementation as the base year for policy implementation. We focus on the estimates of $\beta_{m}$ that indicates the difference in GTFEE between the treatment group and the control group m years away from the benchmark year. The meanings of other variables in model (2) remain the same as in model (1).

### 4.2. Variables

#### 4.2.1. Dependent Variable

We constructed firms’ green total factor energy efficiency (GTFEE) as the dependent variable. Compared with SFEE, GTFEE can reflect energy economic system efficiency more comprehensively and effectively. Referring to Wu et al. [14] and Gao et al. [15], we use the undesirable-SBM model to calculate the GTFEE of firms. The undesirable-SBM model is proposed by Tone [48], and it belongs to one of the DEA-derived models. Compared with the traditional DEA model, the undesirable-SBM model not only avoids the bias caused by radial and angular measures but also takes into account the influence of undesirable output factors in the production process, which better reflects the essence of efficiency evaluation.

To be specific, we assume that each firm is a decision-making unit (DMU), the number of which is N. We suppose each decision-making unit has M inputs, S_1_ expected outputs and S_2_ unexpected outputs, which can be represented in the form of matrices $X=(x_{ij})\in R_{m\times n},\;Y^{g}=\left(y_{ij}^{g}\right)\in R_{s1\times n},\;Y^{b}=\left(y_{ij}^{b}\right)\in R_{s2\times n}$. Specifically, $s^{-}\in R_{m}$, $s^{g}\in R_{s1}$, and $s^{b}\in R_{s2}$ are the corresponding relaxation vectors of input, expected output, and unexpected output, respectively. In addition, λ is the weight vector. The basic calculation formula is as follows:

(3) $$\min p^{\prime }=\frac{1-\frac{\left(\frac{1}{m}\right)\sum_{i=1}^{m}s_{i}^{-}}{x_{i0}}}{1+\frac{1}{s_{1}+s_{2}}\left(\frac{\sum_{r=1}^{s_{1}}s_{r}^{g}}{y_{r0}^{g}}+\frac{\sum_{r=1}^{s_{2}}s_{r}^{b}}{y_{r0}^{b}}\right)}\;S.t.\;x_{0}=X\lambda +s^{-}\;\;\;\;\;\;\;\;\;\;y_{0}^{g}=Y^{g}\lambda -s^{g}\;\;\;\;\;\;\;\;\;y_{0}^{b}=Y^{b}\lambda -s^{b}\;\lambda \geq 0,s^{-}\geq 0,s^{g}\geq 0,s^{b}\geq 0$$

The measurement of GTFEE mainly includes the firm’s inputs, expected outputs and unexpected outputs. The specific index selection and its measurement are shown in Table 2. The inputs are divided into capital stock, energy consumption, and labor force. They are denoted by the total capital stock, total energy consumption, and the total number of employees of each firm. The total output value of the enterprise are selected to measure desirable output. Using energy creates maximum expected output, it also requires control to minimize environmental pollution. Therefore, we select each industrial firm’s total industrial wastewater discharge, total sulfur dioxide emission and total industrial solid waste emissions to measure the undesired output. The data from the Chinese Industrial Enterprise Database and the Chinese Industrial Enterprise Pollution Database from 2003 to 2013.

#### 4.2.2. Independent Variable

We use $RCS_{it}$ to represent the independent variables, where *i* represents the city and *t* represents the year. $RCS_{it}$ is the dummy variable. We set RCS to 1 if the city *i* has implemented RCS in year *t* and 0 otherwise.

#### 4.2.3. Mechanisms Variable

Energy structure: regarding Bu et al. [13], we use coal consumption, fuel oil consumption, and clean gas consumption to reflect the change in energy structure. The RCS increases the pollution discharge cost of enterprises, which in turn prompts enterprises to optimize their energy structure [49]. The optimization of energy structure can improve GTFEE [43].

Technology innovation: referring to Gao et al. [2], we use firm’s R&D expenditure as a measure of firm’s technological innovation. The RCS can force firms to engage in green innovation activities [8,44]. Additionally, technological innovation is an important way to improve GTFEE [15].

#### 4.2.4. Other Control Variables

Referring to Bu et al. [13] and Huang et al. [12], the control variables are as follows: (1) Firm age (FA), measured by subtracting the year of establishment from the sample year and adding 1, and then taking the logarithm; (2) firm size (FZ), measured by the logarithm of the firm’s total assets; (3) firm profit (FP), measured by the logarithm of the firm’s total profit; (4) firm debt ratio (FR), measured as the ratio of total liabilities to total assets; (5) technological innovation (TI), measured by the logarithm of the enterprise’s R&D expenditure; (6) whether the enterprise is an exporter, a dummy variable, 1 when the enterprise has an export business, 0 otherwise; (7) whether the enterprise is a state-owned enterprise, a dummy variable, 1 when the enterprise is a state-owned enterprise, 0 otherwise. Among them, the selection of control variables such as firm age (FA), firm size (FZ), technological innovation (TI), whether the enterprise is an exporter, and whether the enterprise is a state-owned enterprise is mainly based on Bu et al. [13]. The selection of the two control variables of firm profit (FP) and firm debt ratio (FR) is mainly based on Huang et al. [12]. We expect that the control variables of firm age (FA), firm size (FZ), firm profit (FP), technological innovation (TI), and whether the enterprise is an exporter all have positive effects on GTFEE, while the two control variables of whether the enterprise is a state-owned enterprise and firm debt ratio (FR) all have negative effects on GTFEE. Descriptive statistics of the above variables are shown in Table 3.

### 4.3. Data Sources

The enterprise pollution emission data in this paper are obtained from the environmental statistics database of Chinese industrial enterprises, and the enterprise-level economic indicators are obtained from the database of Chinese industrial enterprises. In recent years, many scholars have used the above two databases to study Chinese enterprises’ economic and environmental pollution [50,51]. Meanwhile, this paper merges the above two databases by using the legal person code and enterprise name of enterprises as matching variables. 

The sample interval in this paper is set to the period 2003-2013. The cities in the Yangtze River Delta region, where the Taihu Lake basin is located, have comparable levels of economic development, close geographical proximity, and connected governance waters. To avoid the influence of cross-regional and cross-basin factors, referring to Wang et al. [52], we restrict our sample to firms in the Yangtze River Delta region. 

The data related to the implementation of the river chief system are compiled from the documents of each municipal government. As of 2013, 22 of the 41 cities in the Yangtze River Delta region have initiated the “river chief system” policy.

Finally, this paper refers to the standard processing method of the Chinese industrial enterprise database [53] and performs data cleaning on this database: (1) eliminating enterprises with less than 8 employees; (2) eliminating enterprises with non-positive values of gross industrial output value, current assets, fixed assets, and product sales revenue; (3) eliminating enterprises with current assets or fixed assets larger than total assets; (4) excluding enterprises with only one-year observation; (5) excluding enterprises with an asset-liability ratio less than 0. We finally obtained 35,287 valid data during the sample period.

## 5. Empirical Results

### 5.1. Baseline Model Results

Table 4 shows the basic regression results of the impact of RCS implementation on firm’s GTFEE. Column (1) does not add control variables and only controls for firm fixed and year fixed effects. Column (2) adds firm-level control variables to column (1). The estimated coefficient β of the independent variable RCS in columns (1) and (2) is significantly positive at the significance level of 1%, indicating that implementing the river chief system significantly improves the GTFEE of enterprises. In order to eliminate the influence of city and industry factors on the results, columns (3) and (4) add city fixed effects and industry fixed effects based on columns (1) and (2), respectively. The results of columns (3) and (4) show that implementing the river chief system significantly improves the GTFEE of enterprises at the significance level of 1%. Based on column (4), the estimated coefficient β of the independent variable RCS is 0.053, indicating that after implementing the river chief system in cities, the GTFEE of enterprises increases by about 5.3%. This finding is consistent with the conclusion of much-existing literature and confirms the positive effect of environmental regulation on energy efficiency [15,16,17,19]. This result is consistent with Hypothesis 1.

In terms of control variables, the estimated coefficient of LnFA is significantly positive, indicating that the older the firm, the higher the GTFEE of the firm. The estimated coefficient of LnFZ is significantly positive, indicating that an increase in firm size can increase a firm’s GTFEE. The estimated coefficient of FR is significantly negative, indicating that corporate debt ratio has a negative impact on GTFEE. The estimated coefficient of LnFP is significantly positive, indicating that the more profit the enterprise has, the higher the GTFEE of the enterprise. The estimated coefficient of LnTI is significantly positive, indicating that the improvement of corporate innovation capabilities can improve corporate GTFEE. The estimated coefficient of exporter enterprise is significantly positive, indicating that the export of enterprises is conducive to the improvement of GTFEE. The estimated coefficient of state-owned enterprise or not is significantly negative, indicating that state-owned enterprises have lower GTFEE. The regression results of the above control variables are in line with our expectations.

### 5.2. Robustness Test

#### 5.2.1. Parallel Trend Test

**Figure 1:**
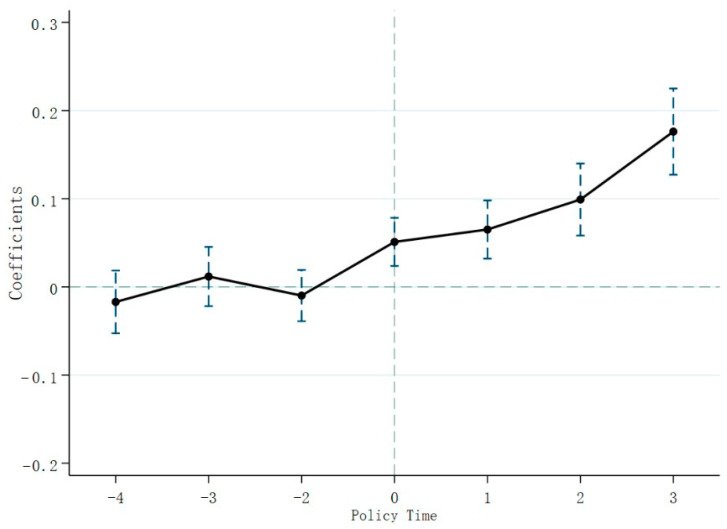
Parallel trend test.

A prerequisite for using the multiple DID model is that before the implementation of the policy event, the samples of the experimental group and the control group share a common trend of change. We construct the following model (2) to verify whether the samples satisfy the parallel trend assumption. The results in Figure 1 show that $\beta_{-4}$, $\beta_{-3}$, and $\beta_{-2}$ are not significant at the 5% significance level. This result shows that before the implementation of the river chief policy, there is no significant difference in GTFEE between the experimental group and the control group, that is, the parallel trend test is valid. At the same time, since the RCS policy started in 2008, we took the previous year, 2007, as the base period and deleted the data for that year. We found the coefficients of $\beta_{0}$, $\beta_{1}$, $\beta_{2}$, and $\beta_{3}$ are all significantly positive at the significance level of 5%, indicating that RCS can significantly improve GTFEE, and the improvement of GTFEE is due to RCS rather than the exante trend.

#### 5.2.2. Placobo Test 

**Figure 2:**
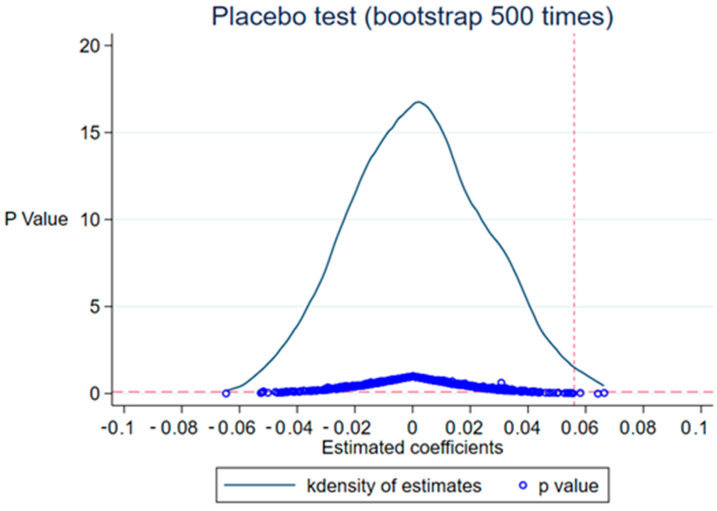
Randomly selected experimental groups.

To confirm that the findings of the baseline regression were not due to random factors, referring to Bradley et al. [54], we randomly selected 22 cities from all sample cities as the pseudo-experimental group (cities with river chief system implementation) for the placebo test. Specifically, in each regression, 22 cities were randomly selected from the sample of 42 cities as the pseudo-treatment group, and these cities were assumed to be the cities where the river chief system was implemented. The remaining cities were assumed to be the pseudo-control group, which did not implement the river manager system. Since the pseudo-treatment group is randomly selected, the pseudo-river length system variable $RCS_{it}^{fake}$ generated by the pseudo-treatment group does not significantly affect GTFEE, i.e., the estimated coefficient $\beta_{fake}$ = 0. That is, the regression coefficient $\beta_{fake}$ will not be significantly different from 0 if it is not a random factor that leads to the findings of the baseline regression. Conversely, it indicates that the findings of this paper are affected by the influence of random factors. Additionally, to avoid the interference of other small probability events on the estimation results, we repeat the above process 500 times. Figure 2 reports the P-value distribution and the probability density of the estimated coefficient $\beta_{fake}$ for the 500 randomly selected treatment groups. The results in Figure 2 show that the mean value of the estimated coefficient $\beta_{fake}$ is close to 0 and concentrated around 0 in an approximately normal distribution, and the true estimated coefficient 0.053, represented by the vertical line in Figure 2, is at the edge of the normal distribution in Figure 2. This result implies that the pseudo-rivermaster variable $RCS_{it}^{fake}$ does not significantly affect GTFEE and the main regression results of this study are not influenced by random factors.

#### 5.2.3. Advance Policy Implementation Time

To exclude the influence of some potential factors on firms’ GTFEE, we construct a counterfactual test by referring to Topalova [55]. We firstly advance the implementation time of RCS in each city by one year, two years and three years, respectively, and then test whether the variable of the river chief system $RCS_{it}^{ad}$ with the implementation time in advance still has an improving effect on GTFEE. If the estimated coefficient $\beta_{ad}$ of the river chief variable $RCS_{it}^{ad}$ is not significant, it can be proved that RCS does have a significant promoting effect on firms’ GTFEE. Otherwise, it means that some underlying unobservables will also improve firms’ GTFEE, not just the result of RCS implementation. Columns (1), (2), and (3) of Table 5 show the regression results of policies one year, two years, and three years ahead, respectively. The results show that after the implementation time of RCS is advanced, the estimated coefficient $\beta_{ad}$ of the river chief variable $RCS_{it}^{ad}$ is not significant. Therefore, the influence of other potential factors on GTFEE can be excluded, which proves the reliability of the benchmark regression results.

#### 5.2.4. Replace Energy Efficiency Measures

Referring to Bu et al. [13], we also use single factor energy efficiency (SFEE) as a measure of energy efficiency in the paper. Therefore, to ensure that the dependent variable energy efficiency measures do not affect the main findings of this paper, we use SFEE as a measure of energy efficiency for robustness testing. The results in column (4) of Table 5 show that RCS still significantly improves SFEE, proving the robustness of the conclusions of this paper.

#### 5.2.5. Propensity Score Matching Method (PSM)

To alleviate the possible endogeneity between the river chief system and GTFEE, drawing on Wang et al. [52], we adopt the propensity score matching method (PSM) to deal with the problem of possible sample selection bias. Specifically, we choose the nearest neighbor matching with a 1:1 matching ratio. At the same time, since the river chief system is implemented gradually, for new entrants to the policy treatment, we find a control group of firms for them year by year from those that have never been affected by the river chief system during the sample period. Finally, we keep all the successfully matched enterprises as the next DID regression sample. The results in column (5) of Table 5 show that RCS still positively affects GTFEE at the 1% level of significance. It can be seen that the results of the regression by the PSM-DID method are also highly consistent with the basic conclusions of this paper.

## 6. Further Analysis

### 6.1. Mechanism Test

This paper explores how the RCS can improve the company-level GTFEE by optimizing the energy structure and promoting technological innovation. This part empirically tests the reliability of the two influence paths. First, we replace the dependent variables in model (1) with the logarithms of coal consumption, fuel oil consumption, and clean gas consumption, respectively, and then perform a regression analysis to test whether RCS optimizes the energy mix. The results in column (1) of Table 6 show that RCS significantly reduces the use of highly polluting and energy-intensive coal at the 1% level. The results in columns (2) and (3) of Table 6 show that RCS contributes significantly and positively to using low-polluting and low-energy fuel oil and clean gas. These results in columns (1) to (3) show that RCS can improve the energy mix of companies by reducing the misuse of high-polluting and energy-intensive energy sources, which in turn promotes the improvement of firms’ GTFEE. This result proves the effectiveness of the energy mix mechanism, which verifies Hypothesis 2. Second, we replace the dependent variable in model (1) with the logarithm of R&D costs and then perform a regression analysis to test the effect of RCS on technological innovation. The results in column (4) of Table 6 show that RCS significantly increases firms’ R&D expenditures at the 1% level. This result is consistent with Hypothesis 3.

### 6.2. Heterogeneity Analysis

The difference in enterprise characteristics may affect the implementation effect of RCS. We will analyze the heterogeneity from three aspects: enterprise size, whether the enterprise is an exporter, and whether the enterprise is in a heavily polluting industry.

Heterogeneity of enterprise size: column (1) of Table 7 shows the regression results of firm size heterogeneity. The estimated coefficient of the interaction term RCS×Scale in column (1) is significantly positive at the 1% level, indicating that the larger the enterprise size, the greater the effect of RCS on the promotion of GTFEE. The possible explanation is that China’s environmental policies are often characterized by “focusing on the large while releasing the small”, thus achieving greater policy effects with smaller administrative costs [52]. Large enterprises feel stronger environmental regulations than small enterprises and are more motivated to increase their GTFEE.

The heterogeneity of whether the firm is an exporting firm: column (2) of Table 7 shows the regression results of firm export heterogeneity. The interaction term RCS×Export in column (2) is significantly positive, indicating that RCS has a greater promoting effect on GTFEE in exporting firms than in non-exporting firms. The possible explanation is that compared with non-exporting enterprises, exporting enterprises need to face stringent international green export standards [13]. After the implementation of RCS, exporting enterprises pay more attention to environmental issues than non-exporting enterprises and assume corresponding environmental responsibilities.

The heterogeneity of whether the enterprises are in the heavy polluting industries: column (3) of Table 7 is the heterogeneous regression result of whether the firm is in a heavily polluting industry. The interaction term RCS×Heavy Pollution in column (3) was significantly positive, indicating that RCS had a greater promoting effect on GTFEE for enterprises in heavy polluting industries than for enterprises in non-heavy polluting industries. This result shows that enterprises in heavily polluting industries are more affected by the river chief system.

## 7. Conclusions and Implications

This paper empirically examines the impact of the river chief system ( RCS) on firms’ GTFEE using data from Chinese industrial firms from 2003 to 2013. First, we measured the GTFEE of enterprises for the first time using data from Chinese industrial enterprises. Second, we used the establishment of the RCS by local governments as a quasi-natural experiment to quantitatively analyze the impact of RCS on GTFEE using a multiple DID approach. The study results are as follows: (1)the RCS can significantly improve GTFEE. The GTFEE of firms increases by about 5.3% after implementing the river chief system. The results of a series of robustness tests indicate that the findings of this study are robust; (2) the mechanism test finds that RCS can improve GTFEE by optimizing energy structure and promoting technological innovation; (3) the policy effects of the RCS show heterogeneity in terms of firm size, whether the firm exports, and whether the firm is in a heavily polluting industry. Specifically, the larger the firm’s size, the greater the effect of RCS on GTFEE enhancement. The promotion effect of RCS on the GTFEE of exporting firms is greater than that of non-exporting firms. The promotion effect of RCS on the GTFEE of firms in heavy pollution industries is greater than that of firms in non-heavy pollution industries.

This paper makes the following policy recommendations. First, the RCS is a major innovative environmental policy implemented by local governments in China and, for the first time, includes environmental protection responsibilities in the assessment and promotion of local officials. Our study finds that RCS can significantly improve firms’ GTFEE, which helps the Chinese government further improve its environmental protection management system. On the one hand, China’s central government should fully decentralize and encourage local governments to promote institutional innovation in environmental governance and become the mainstay of environmental governance. On the other hand, the Chinese government needs to include environmental responsibilities in the performance assessment of local officials when formulating other command-based environmental policies. Second, we find that RCS improves GTFEE by optimizing the energy mix. Coal accounts for more than 94% of China’s energy reserves, while oil and natural gas account for only about 6%, and this status quo determines that China’s energy consumption mix is dominated by coal [17]. Therefore, the Chinese government should actively promote the optimization of China’s energy structure by increasing the import of oil and natural gas on the one hand, and the acceleration of the development of new energy industries on the other hand, to promote the transformation of the energy structure from coal to clean energy and renewable energy. Third, the RCS can improve GTFEE through technological innovation; therefore, the Chinese government should implement various subsidy policies and increase financial support for enterprises to carry out green innovation activities to encourage them to research and develop green technologies.

Although this paper comprehensively analyzes the relationship between RCS policy and enterprise energy efficiency, there are still some limitations. For example, it may omit the promotion pressure of officials related to RCS policy, political connections, and other factors affecting enterprise energy efficiency. Although it is impossible to explore exhaustively the factors affecting enterprise energy efficiency, this may be a future research direction. In addition, in future studies, it is also an important theoretical and practical issue worth discussing to explore how RCS policy, as environmental regulation, influences the profits of enterprises. This is because the coordination of the relationship between environmental protection and enterprise development is the internal driving force to motivate enterprises to spontaneously save energy and reduce emissions.

## Figures and Tables

**Table 1:** List of cities implementing the river chief system.

Implementation Year	Implementation Cities
2007	Wuxi City
2008	Yixing City, Changzhou City, Suzhou City
2009	Yancheng City, Huaian City, Taizhou City
2010	Yangzhou City
2011	Zhenjiang City, Nantong City, Lianyungang City, Taizhou City, Ningbo City, Jiaxing City
2012	Shaoxing City, Hefei City
2013	Xuzhou City, Nanjing, Hangzhou City, Huzhou City, Quzhou City, Huangshan City

**Table 2:** Construction of indicators for the GTFEE measure of enterprises.

Category	Indicators	Measurement
Input	Labor force (L)	Number of employment in enterprises
	Capital stock (K)	Fixed assets of the enterprise
	Energy consumption (EU)	Coal consumption of enterprises
		Fuel oil consumption of enterprises
		Clean gas consumption of enterprises
Expected output	Industrial output	The total output value of the enterprise
Unexpected output	Industrial wastewater	Wastewater emissions of enterprises
	Industrial sulfur dioxide	Sulfur dioxide emissions of enterprises
	Industrial soot	Solid waste emissions of enterprises

**Table 3:** Descriptive statistics.

Variable	Obs	Mean	Std. Dev.	Min	Max
Firms’GTFEE	35,287	0.149	0.058	0.013	1.000
LnFA	35,287	2.354	0.648	0.000	5.011
LnFZ	35,287	10.974	1.191	7.339	15.332
FR	35,287	0.620	0.250	0.000	5.554
lnFP	35,287	6.297	6.020	−12.742	14.385
lnTI	35,287	0.317	1.387	0.000	10.721
Exporter enterprise or not	35,287	0.035	0.185	0.000	1.000
State-owned enterprise or not	35,287	0.411	0.492	0.000	1.000

**Table 4:** The benchmark regression results.

	(1)	(2)	(3)	(4)
RCS	0.049 ***	0.050 ***	0.052 ***	0.053 ***
	(0.010)	(0.011)	(0.009)	(0.011)
LnFA		0.025 *		0.025 *
		(0.013)		(0.013)
LnFZ		0.115 ***		0.113 ***
		(0.010)		(0.010)
FR		−0.053 **		−0.050 **
		(0.023)		(0.023)
LnFP		0.002 ***		0.002 ***
		(0.001)		(0.001)
LnTI		0.018 ***		0.017 ***
		(0.003)		(0.003)
Exporter enterprise or not		0.070 ***		0.068 ***
		(0.011)		(0.011)
State-owned enterprise or not		−0.089 **		−0.091 **
		(0.043)		(0.043)
Constant	−4.705 ***	−2.468 ***	−4.706 ***	−2.491 ***
	(0.004)	(0.114)	(0.004)	(0.114)
Firms FE	Yes	Yes	Yes	Yes
Year FE	Yes	Yes	Yes	Yes
City FE	No	No	Yes	Yes
Industry FE	No	No	Yes	Yes
N	35287	35287	35287	35287
R^2^	0.805	0.810	0.809	0.814

Notes: The value in parentheses are standard errors clustered at the firm level; ***, **, and * represent that coefficients are significant at the 1%, 5%, and 10% levels, respectively.

**Table 5:** Robustness test.

	(1)	(2)	(3)	(4)	(5)
	RCS_1	RCS_2	RCS_3	SFEE	PSM
RCS	0.021	0.003	0.011	0.046 **	0.062 ***
	(0.019)	(0.017)	(0.019)	(0.023)	(0.012)
Control	Yes	Yes	Yes	Yes	Yes
Firms FE	Yes	Yes	Yes	Yes	Yes
Year FE	Yes	Yes	Yes	Yes	Yes
City FE	Yes	Yes	Yes	Yes	Yes
Industry FE	Yes	Yes	Yes	Yes	Yes
N	35287	35287	35287	35287	33058
R^2^	0.814	0.814	0.814	0.825	0.718

Notes: The value in parentheses are standard errors clustered at the firm level; *** and ** represent that coefficients are significant at the 1% and 5% levels, respectively.

**Table 6:** Mechanism tests.

	(1)	(2)	(3)	(4)
	Lncoal	Ln fuel	Lngas	Ln R&D expenses
RCS	−0.067 ***	0.085 **	0.075 **	0.080 ***
	(0.019)	(0.038)	(0.037)	(0.025)
Control	Yes	Yes	Yes	Yes
Firms FE	Yes	Yes	Yes	Yes
Year FE	Yes	Yes	Yes	Yes
City FE	Yes	Yes	Yes	Yes
Industry FE	Yes	Yes	Yes	Yes
N	35,287	35,287	35,287	35,287
R^2^	0.715	0.723	0.737	0.562

Notes: The value in parentheses are standard errors clustered at the firm level; *** and ** represent that coefficients are significant at the 1% and 5% levels, respectively.

**Table 7:** Heterogeneity analysis.

	(1)	(2)	(3)
	Firm size	Export	Heavy polluting industries
RCS	0.044 ***	0.117 ***	0.007
	(0.015)	(0.014)	(0.014)
RCS×Scale	0.004 ***		
	(0.001)		
RCS×Export		0.127 ***	
		(0.017)	
RCS×Heavy Pollution			0.101 ***
			(0.018)
Control	Yes	Yes	Yes
Firms FE	Yes	Yes	Yes
Year FE	Yes	Yes	Yes
City FE	Yes	Yes	Yes
Industry FE	Yes	Yes	Yes
N	35,287	35,287	35,287
R^2^	0.842	0.814	0.814

Notes: The value in parentheses are standard error clustered at the firm level; *** represent that coefficient is significant at the 1% levels.

## Data Availability

Not applicable.
